# Supplementary material for: “We’re Not Going to Be as Prepared”: A Qualitative Study of Healthcare Trainees’ Experiences after One Year of the COVID-19 Pandemic
Source: Int J Environ Res Public Health. 2023 Feb 27;20(5):4255. doi: 10.3390/ijerph20054255 (PMC10002117; doi:10.3390/ijerph20054255)
Supplement: Supplementary file 1 [file ijerph-20-04255-s001.zip › Supplementary File S1 COREQ_Consolidated criteria for reporting qualitative studies .pdf]

Supplementary file S1. Consolidated criteria for reporting qualitative studies (COREQ): 32-item checklist

Topic and Item No.	Guide Questions/Description	Response
**Domain 1: Research team and reflexivity**		
Personal Characteristics		
1. Interviewer/facilitator	Which author/s conducted the interviews?	Alex Brewer (AB)
2. Credentials	What were the researcher's credentials? E.g. PhD, MD	HB: PhD<br>NC: PhD<br>AB: MSc
3. Occupation	What was their occupation at the time of the study?	HB: health psychologist/university academic;<br>NC: applied psychologist / health researcher<br>AB: project manager / researcher / MSc student
4. Gender	Was the researcher male or female?	2 female, 1 male
5. Experience and training	What experience or training did the researcher have?	Mixed-methods researchers. Trained in research ethics, research integrity, interview skills.
Relationship with participants		
6. Relationship established	Was a relationship established prior to study commencement?	Researchers met the participants during recruitment.
7. Participant knowledge of the interviewer	What did the participants know about the researcher? e.g. personal goals, reasons for doing the research	Participants knew that AB was a researcher conducting the project as part of his MSc, and that HB and NC were university staff and researchers.
8. Interviewer characteristics	What characteristics were reported about the interviewer/facilitator? e.g. Bias, assumptions, reasons and interests in the research topic	Participants knew that the interviewer was interested in the wellbeing of healthcare trainees.
**Domain 2: Study design**		
Theoretical framework		
9. Methodological orientation and Theory	What methodological orientation was stated to underpin the study? e.g. grounded theory, discourse analysis, ethnography, phenomenology, content analysis	Deductive and inductive thematic analysis
Participant selection		
10. Sampling	How were participants selected? e.g. purposive, convenience, consecutive, snowball	Convenience sample during a set recruitment period, March-May 2021.
11. Method of approach	How were participants approached? e.g. face-to-face, telephone, mail, email	Study promoted via social media (student and healthcare Facebook groups and official Twitter sites), and student-facing mailings, and subsequent contact was by email.
12. Sample size	How many participants were in the study?	12 participants took part in individual qualitative interviews

13. Non-participation	How many people refused to participate or dropped out? Reasons?	No participants actively refused to participate or withdrew following consent.
Setting		
14. Setting of data collection	Where was the data collected? e.g. home, clinic, workplace	Data were collected online (video-conferencing platform)
15. Presence of non-participants	Was anyone else present besides the participants and researchers?	Interviews: No.
16. Description of sample	What are the important characteristics of the sample? e.g. demographic data, date	Age, gender, subject discipline, year of study, work-related covid-19 exposure during the pandemic (in health or social care environments)
Data collection		
17. Interview guide	Were questions, prompts, guides provided by the authors? Was it pilot tested?	Question guide was developed by the project team and discussed with a healthcare trainee who was not a participant in the study.
18. Repeat interviews	Were repeat interviews carried out? If yes, how many?	No repeat interviews.
19. Audio/visual recording	Did the research use audio or visual recording to collect the data?	Interviews were audio-recorded.
20. Field notes	Were field notes made during and/or after the interview?	No.
21. Duration	What was the duration of the interviews or focus group?	Interviews lasted between 20 and 37 minutes, with an average time of 33 minutes.
22. Data saturation	Was data saturation discussed?	Recruitment continued until the dataset was deemed to hold sufficient information power.
23. Transcripts returned	Were transcripts returned to participants for comment and/or correction?	No, due to time constraints.
**Domain 3: analysis and findings**		
Data analysis		
24. Number of data coders	How many data coders coded the data?	One coded the data, a second researcher checked the codes.
25. Description of the coding tree	Did authors provide a description of the coding tree?	No, however initial coding was informed by the interview guide, and coding was continuously refined.
26. Derivation of themes	Were themes identified in advance or derived from the data?	Initial coding framework was derived based on a prior study, which was refined based on the data and generated codes (combined deductive-inductive approach).
27. Software	What software, if applicable, was used to manage the data?	None.
28. Participant checking	Did participants provide feedback on the findings?	No, due to time constraints.
Reporting		
29. Quotations presented	Were participant quotations presented to illustrate the themes or findings?	Yes, quotes included and identified with participant ID.

	Was each quotation identified? e.g., participant number	
30. Data and findings consistent	Was there consistency between the data presented and the findings?	Yes.
31. Clarity of major themes	Were major themes clearly presented in the findings?	Yes.
32. Clarity of minor themes	Is there a description of diverse cases or discussion of minor themes?	Yes.
