# Supplementary material for: “We’re Not Going to Be as Prepared”: A Qualitative Study of Healthcare Trainees’ Experiences after One Year of the COVID-19 Pandemic
Source: Int J Environ Res Public Health. 2023 Feb 27;20(5):4255. doi: 10.3390/ijerph20054255 (PMC10002117; doi:10.3390/ijerph20054255)
Supplement: Supplementary file 1 [file ijerph-20-04255-s001.zip › Supplementary file S2_Interview Topic Guide.pdf]

Interview Topic Guide

Introduction

1. If we could start with a little background about you – what is your current area of study? (prompts: how long have you been on the (insert course as appropriate)).

Impact of COVID on wellbeing

2. Can you tell me a bit about what was it like being a healthcare student during Covid-19; what have been the emotional highs and lows of the pandemic, looking back from where you are now? Has the duration of the pandemic impacted on your wellbeing? Any negative or positive effects?
3. Have you had to self-isolate for a period of time as a result of exposure/potential exposure? (once or more than once? If so, when? Were there any particular impacts of isolating (once or more than once) e.g. on you or others? Mood, social behaviour). Seek examples at different stages of the pandemic.

Impact of COVID on University studies and learning

4. Has any aspect of your learning been impacted during the past year?
5. Can you tell me a bit about your experiences during the transition to online learning? What has worked well, or less well? What has helped you adapt to this format? (prompts: ask for specific examples/support systems).
6. Have you had any particular concerns about your studies given the duration of the pandemic (prompts: academic learning, opportunities for clinical skills development, examinations, concerns relating to the future – qualifying and future jobs?)
7. Have you spent any time on placement in the NHS during the pandemic? What have your experiences been? Are there any specific points that have been more, or less challenging? (seek stories, examples)
8. Have you had any experience of being asked to act as in any other role in the NHS during the pandemic (e.g., healthcare assistant, support roles) at any point during the pandemic? What was your experience of this?

Support for healthcare students

9. What supports have been available to you during the pandemic? (e.g., family, friends / peers, University, NHS mentors / colleagues or systems). What has been more or less helpful?
10. What kind of support would you like to see offered to healthcare students as a result of your experiences during Covid-19? (By the University, or on clinical placements).
