# Supplementary material for: “We’re Not Going to Be as Prepared”: A Qualitative Study of Healthcare Trainees’ Experiences after One Year of the COVID-19 Pandemic
Source: Int J Environ Res Public Health. 2023 Feb 27;20(5):4255. doi: 10.3390/ijerph20054255 (PMC10002117; doi:10.3390/ijerph20054255)
Supplement: Supplementary file 1 [file ijerph-20-04255-s001.zip › Supplementary file S3_Conceptual Model.pdf]

Supplementary file S3. Conceptual model for mitigating the impacts of COVID-19 on health and care workers (as seen in Blake et al, 2021)

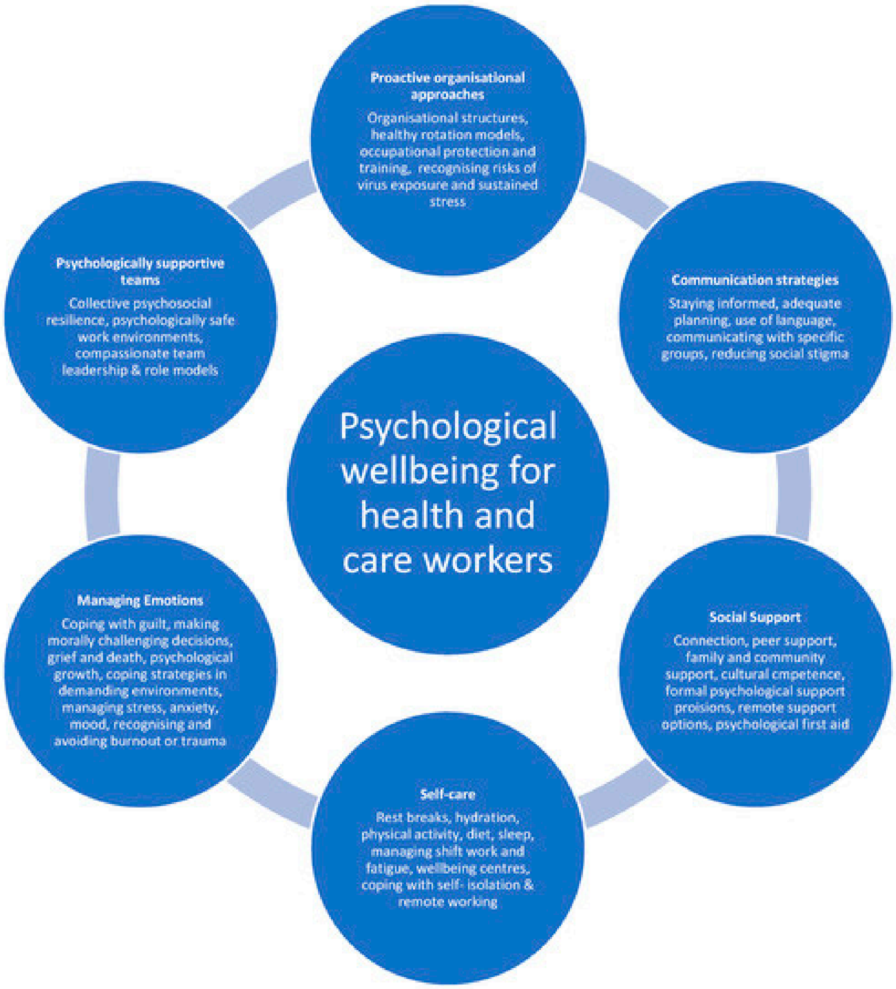

Source:

Blake, H.; Mahmood, I.; Dushi, G.; Yildirim, M.; Gay, E. Psychological Impacts of COVID-19 on Healthcare Trainees and Perceptions towards a Digital Wellbeing Support Package. Int. J. Environ. Res. Public Health 2021, 18, 10647. https://doi.org/10.3390/ijerph182010647
